# Out- Of- Pocket health expenditure and household consumption patterns in Benin: Is there a crowding out effect?

**DOI:** 10.1186/s13561-023-00429-8

**Published:** 2023-03-27

**Authors:** Hilaire Gbodja Houeninvo, Venant Cossi Celestin Quenum, Melain Modeste Senou

**Affiliations:** 1 University of Abomey Calavi, Calavi, Benin; 2 African Economic Research Consortium, Nairobi, Kenya; 3 Economics, African Centre of Excellence for Inequality Research, Cape Town, South Africa

**Keywords:** Health shocks, Consumption patterns, Out of Pocket, Crowding out effect, SURE, Benin, O12 I13

## Abstract

Health shocks are common and have serious consequences for households in developing countries where health insurance is lacking. In this study, we examine whether out-of-pocket health expenditures crowd out household consumption of non-healthcare necessities, such as education items in Benin using a sample of 14,952 households from the global vulnerability and food security analysis survey. We estimated a system of conditional Engel curves with three stage least squared (3SLS) and seemingly unrelated regression (SURE) for seven categories of goods using the Quadratic Almost Ideal Demand System (QUAIDS) in the form of budget shares corresponding to proportions of total non-health expenditure. Findings show that out of pocket health expenditure leads households to spend more on health care that in fine crowd out expenditure in other necessity goods such as education item. These findings highlight the need for social protection programs to mitigate the impact of health shocks on vulnerable households in Benin.

## Introduction

Households in developing countries often face a number of shocks that negatively affect their well-being outcome including incomes, health, education [34, 57]. A shock is defined as a random event likely to reduce the well-being of the individual, namely an illness, a death or a flood, making him more vulnerable to chronic poverty (Dercon et al., 2005; [5, 10]. From the literature, there are two main categories of shocks including idiosyncratic shocks or shocks specific to each household such as illness, death, loss of employment and covariate shocks or shock affecting several households in the same geographic area such as a flood or an epidemic (Krueger et al., 2016,Dercon et al., 2005; [10]. The idiosyncratic shock (mainly health shock) has a wider effect on the vulnerability of households because of their sensitivity to food prices, household size, high loans, labor supply, productivity, human capital accumulation etc. [3, 60]. The economic vulnerability of households is usually appreciated with indicators including food expenditures as well as health and education spending (Krueger et al., 2016,[60]. In addition, economic vulnerability depends on the profile of households. We observe that the households that have the largest share of their budget devoted to food are mainly those living with assistance be it from friends, parents or even from the government (WFP, 2017). As a result, expenditures on food and education in the context of health shock deserve much investigation because of their significant roles in household welfare [47]. This is because the occurrence of serious illness can leave households to cope with large medical expenditures that may be catastrophic, especially when access to health insurance is poor and when publicly funded health care programs are inadequate or absent [14].

Two main categories of coping strategies have emerged from the literature. There exists ex-ante strategies such as precautionary savings, insurance products or asset accumulation and ex-post strategies, which depend on the period of action (Dercon et al., 2005; [19]. In most cases, households in situations of unexpected shocks like health shock adopt the ex-post strategy, which consists in experiencing first the shock, and then looking for adequate measures according to their ability to control the effects of the shock. For instance, households circumvent health shocks by looking for alternative sources of financing for out-of-pocket health expenditure [22, 52, 60], substituting labor or increasing the working time of household members and reducing expenditure on food and non-food items as well as dropping out children from school [58],WHO, 2007; [3, 20]. For example, in examining the impact of malaria on household consumption in Tanzania, Somi et al. [62] found by estimating Engel curves that household reduce their expenditure on luxury items in response to health care spending. Similarly, Kim & Yang [37], investigating the relationship between catastrophic health expenditures and household incomes and expenditure patterns in South Korea found that all consumption categories, other than health expenditure, were significantly lower in households with catastrophic health expenditures than in those without catastrophic health expenditures. However, increased out of pocket health expenditure is a detrimental way of financing healthcare, as it increases the financial risk and barriers of healthcare access especially for the most vulnerable groups, such as lower socioeconomic status families [32].

In Benin, more than 96% of households affected by a shock suffered a reduction in income and barely 10% of the affected household was able to completely recover. The main idiosyncratic shocks faced by households are health shock, accident or death of a household member, which affected 60.9% of households (WFP, 2017). Indeed, some studies evidenced that the vulnerability of poor to shock in developing countries like Benin may be due to several factors including the low shock coping capacity of households and the absent or ineffective institutional arrangements such as effective social protection programs to cope with shocks [12, 47, 50, 77]. In fact, in 2011, the government set up a national health insurance called the « Régime d’Assurance Maladie Universelle»; however, this national health insurance is not yet effective. This was followed by a number of public and private health insurance scheme including the free caesarean, free malaria care for children from 0 to 5 years old [44] that aimed at to reducing barriers to accessing good-quality healthcare [28]. According to the World Health Organization (WHO), medical fee is a major barrier to the access and utilization of healthcare services. Instead of relying on the out-of-pocket expenditure, WHO recommends that governments adopt the risk-pooling payment approach to realize the social goal of universal coverage of healthcare [12]. Indeed, given its success in a number of emerging countries and some African countries, social protection system including health system financing is seen as a tool that has a mandate to improve the lives of poor people [74]. Financial protection in healthcare has long been recognized as a major policy goal, since it relates to the broader issue of access to adequate and quality healthcare and equity in health status [12, 43].

More recent surveys from the Ministry of health of Benin indicate that only 8.4% of the population is covered by some form of health insurance and 41.6% of the population is living below [44]. According to the poverty line Benins’ National Institute of Statistics and Demography ([INStaD], 2015) and [64],WFP, 2017; ANPS, 2021). While the contribution of the government expenditure on health is 54.2% of the total expenditure on Benin’s health, the financing system is dominated by private finance including OOP payments [27]. Indeed, the share of domestic private health expenditure is still significant (almost 45.8% of current health expenditure) and, in particular, out-of-pocket expenditure comprise more than 89.2% of current private health expenditure, amounting to USD 38.08 (purchasing power parity) per capita in 2017. The share of voluntary health insurance is very limited and stagnant over time (slightly above 5% of current health expenditure). It is well documented that large out-of-pocket spending on medical issues can expose households to catastrophic health expenditure, which can result in poverty [22, 46]. For example, using WFP (2017), Houeninvo [27] suggested that 25.49% of households spent 40% of their resources on healthcare. In that context of absence of effective social protection programs that should help poor cope shocks they face,OOP payments by the household is a key variable in economic policy, especially when considering the poor and vulnerable groups of the population. Therefore, it is urgent to investigate the choice function of households in order to know the alternative household coping strategies in terms of resource allocation to food and non-food items including health and education. In fact, evidences show that most of households desperately reduce their expenditure by cutting down on food budget, remove children from school, send some family members to relatives, and others [51, 52, 77]. However, when children are removed from school the household’s future income generating capacity is affected. In other words, by reducing its budget devoted to food and education, households undercut critical investments in human capital, inhibiting both current and future productivity [34], Kim & Ahn, 2019).

This study seeks to provide answers to the following research questions: How can health expenditure incurred by households affect the consumption patterns of the household? Is human capital jeopardized in the context of health shocks? By doing this, the study contributes to the relevant literature as follows. First, to the best of our knowledge, there is a paucity of empirical evidence on the relationship between OOP health expenditure and household’s consumption and especially in Benin. This is the first study in Benin that focuses the crowding out effect of health expenditure on consumption of non-healthcare necessities. Second, to our knowledge very few recent studies have assessed the crowding out effect of health. Most of the existing studies are either old and unable to evidence the actual crowding out effect of health expenditure [9, 26] or are on the crowding out effect of tobacco [9].in Benin’s context. We then assess the crowding-out effect of out-of-pocket health expenditures on consumption goods including education by estimating conditional Engel curves for 07 categories of goods using a Quadratic Almost Ideal Demand System (QUAIDS) in order to see whether households subject to a shock, are more likely to change their consumption patterns especially their expenditure on education and food. This approach is relevant in the sense that human capital constitutes the backbone of production and sustainable development. Thus, crowding out expenditures on human capital especially education spending would threaten the human capital, and in turn, the economic development.

Findings of this study show that health shocks lead households to spend more in health care that in fine crowd out expenditure in other necessity goods such as education and foods. These results appear as a basis of social protection programs in Benin because the successive social protection programs were designed considering the vulnerability of poor people to health expenditure. In that context, the share of resource allocated to human capital as well as the exposure to shocks could be key indicators that help selected the most exposed to shock.

The rest of the paper is organized as follows: Sect. 2 presents the overview of the social protection in Benin followed by the review of the relevant literature in Sect. 3. Section 4 presents the estimation strategy. Section 5 presents data and sample procedure as well as the descriptive statistics of the variables used in this study. Section 6 discusses the estimation results. Section 7 concludes the study.

## Overview of the social protection in Benin

The Government of Benin has designed some social legislation texts and laws, with a view of protecting the most vulnerable population, however, its implementation may be challenging. These constraints are the high cost of social protection program (evaluate at USD 4,300,134,927.3 for the period 2014–2018 (Ministry of Planning and Development, 2016), the low covering of risk, the mis-targeting of beneficiaries, and the high dependence to external funds (Ministry of Planning and Development, 2016). As a result, the assistance to vulnerable groups remains a great challenge to improving their living conditions (National Development Plan [PND], 2018–2025). Moreover, Benin has a low social protection index (0.21) far behind South Africa, whose index is 0.80, Burkina (0.27) and Senegal (0.23). Similarly, the National Development Plan for Benin indicated that the coverage rate of the social protection is estimated at 8.4% against 17.8% for Africa and 45% worldwide (PND, 2018–2025) Fig. 1.

**Fig. 1:**
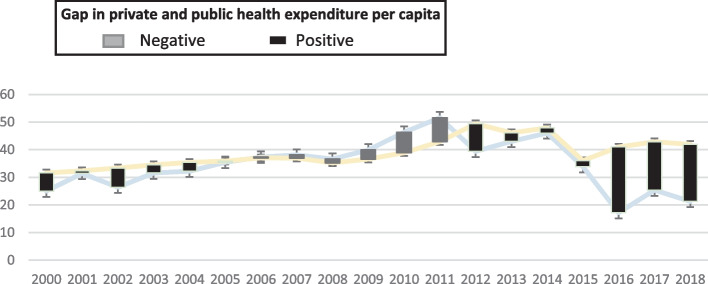
Gap in private and public health expenditure per capita. Source: Authors, 2023

The main weakness of the social protection system in Benin is the exclusion of the poorest and the most vulnerable who mostly earn their income in the informal sector (PND, 2018–2025). Indeed, the weakness of the social security programs in Benin may come from both the demand and supply side. In fact, from demand side, only 6.4% of the economically active population subscribes to the two existing social security funds in Benin (FNRB and CNSS) and 0.1 of them subscribed to RAMU^1^ (PND, 2018–2025). From the supply side, the share of the budget allocated to the social protection has declined between 2015 and 2019 by 18%. This given not only to a reduction in budget allocations to social protection of 39% (from 41.5 to 25.4 billion CFA) but also to the share of the budget in favor of specific social protection interventions^2^ by 33% (from 23.6 billion to 15.9 billion CFA) (UNICEF, 2019). Furthermore, specific social protection account only for 1.3% compared to 6.8% for total social protection. As a result, it worth looking for policies that have more impact on the most vulnerable people including women and children.

## Review of the literature

In the case of health shocks, production is directly affected, implying consequently income loss. Observing or anticipating this shock, the household adopts coping strategies to minimize adverse impacts. If coping strategies are completely effective, then income will be stabilized, and consumption patterns may be largely unaffected. Otherwise, the health shock will lead to changes in household consumption patterns.

Two strands of theory including the life-cycle permanent-income and risk sharing theories inspire studies pertaining to assessing the determinant and impact of shocks on household consumption in developing countries [1, 47, 48]. The former claims that households' current consumption is determined by the present value of lifetime resources whereas the latter also known as consumption insurance claims that idiosyncratic shocks should be absorbed by the cooperation between households within the same risk-sharing network [17, 19, 45]. In fact, Households tend to smooth their consumption over time. But persistent shocks such as health shock may have significant effects on their consumption and poverty as these make households to review their lifetime consumption plans [6]. In the literature, health shocks are defined as an unexpected deterioration in the state of an individual’s health, caused by an illness or injury (WHO, 2007,[49]. Health shocks have both short term and long-term effects. In the short term, health shocks of household can directly reduce their income generating capacity leading to income losses and may induce considerable treatment costs. This can force the household to reduce expenditure on major consumption items such as food or education [23].

There is a growing consensus that access and the quality of health provision as well as socio economic factors could be a major determinant of poor health outcomes in developing countries [16, 66],Houeninvo, 2022). Social and economic determinants have a fundamental influence on health. Therefore, conditions existing within the larger contextual environment are integral to the health of communities and populations [8, 63]. According to the WHO (2013), every aspect of government and the economy has the potential to affect health. Social and economic health risk factors can include person-level attributes, such as sex and gender identification, race and ethnicity, income and wealth, and educational attainment [66]. A second focal area is on the circumstances in which people live. These circumstances include the availability of healthy food and adequate housing, effective public education, community safety, safe employment that pays a living wage. They also include infrastructure for physical activity, diverse transportation options, social and cultural norms for healthy living, social policy that mitigates health shocks as well as political inclusion ensuring social protection for poor. Indeed, exposure to health risk decrease with young people and increases dramatically with older people, as approximately half of lifetime medical expenditure is incurred after the age of 65 [2]. However, concerning the gender, women-headed households are supposed to be more vulnerable to shocks and more particularly to health shock [13]. Higher educational attainment reduces the likelihood of being at risk of health shock [24]. Families generally spread resources more thinly as family size increases. A household would generally have less education expenditure per child or less per capita medical expenditure if the household size rises. At the population level, higher per capita income is associated with better health, and this linkage is robust across many health indicators, including life expectancy, chronic disease burden, and self-rated health status (Marmot, 2005). The association between health and per capita income is evident at multiple levels of observation, from neighborhoods to regional and global levels of analyses.

A health-care finance system in which out-of-pocket expenditure share of total health expenditure is high, and there is a lack of an effective social security net for people, the amount of OOP expenditure can be a large percentage of household income (Houeninvo, 2022, [22, 60]. In such health system, out-of-pocket expenditures lead to catastrophic health expenditures and push some of them into poverty. According to Xu et al. [73], OOP payments is financially catastrophic for a household when it exceeds a certain proportion of a household’s capacity to pay for healthcare, and the family can no longer maintain its customary standard of living. A recent systematic review by Eze et al. [22] suggests that the annual incidence of catastrophic health expenditure in sub-Saharan Africa was high (16.5% for a threshold of 10% of total household expenditure), and the incidence of catastrophic health expenditure increased between 2010 and 2020. In order to cope with household financial vulnerability to health shock, the literature highlight some measures to smooth consumption in response to shocks [19, 46]. In situations where the household has to incur health expenditures exceeding the income of the household, there is going to be a dent in the savings, sometimes to the extent of total exhaustion of savings and ultimately debt generation [31]. Households would as a consequence reduce their consumption and the impact of the shock is felt long after the event has taken place. Moreover, if an earning household member falls sick resulting in further loss of income, the household has no other alternative but to decrease consumption [18]. These households sometimes also have to compromise on their monthly consumption by a considerable amount so that the health expenses of hospitalization are met, and this is mainly because of the absence of any schemes of social protection [5]

Several empirical studies have also evidenced the direct impact of health shock on consumption pattern [12, 23, 70]. For instance, Chantzaras & Yfantopoulos [12] show that health shock inducing higher out of pocket in healthcare exacerbates household poverty. In the same vein, Araujo et al. [5] find that catastrophic health expenditures measured by the 10% and 25% threshold of consumption are largely concentrated among the poorer households compared to the rich one, and more than 4.87% of Brazilians are pushed into poverty due to OOP health care payments. This result is in line with that of Sarker (2021) who argued that OOP cost in Bangladesh is regressive because the poorer income group suffered more and spent up to 35% of their household monthly income on healthcare. In case of Malawi, the incidence of catastrophic health expenditures and the proportion of household pushed in poverty due to out-of-pocket expenditures were low, 1.37% and 1.6% respectively [46]. The authors explained that the low levels of overall incidence of catastrophic health expenditures reflects poor households in ability to afford care due to high costs which forces such households to forgo treatment to avoid the consequences of out-of- pocket health payments. Similarly, Wagstaff & Lindelow [69] find that health shocks are more likely than droughts to cause poor households to cut back their consumption in Laos. These differences may be attributed to the coping strategies, household characteristics as well as social and economic contexts across countries [45, 47]. Indeed, Mu [45] shows that the effectiveness of protection varies with household human capital levels. Gerry & Li [25] show that households with educated heads, smaller household size and living in urban areas are more able to smooth their consumption. Support from relatives, access to credit and social allowances are also shown to be crucial to strengthen households' resilience to shocks.

However, very few studies have analyzed the effect of shocks on household education expenditure. Escobal et al. [21] show that shocks do not have significant impacts on school dropout rates but negatively affect household education expenditure in Peru. In contrast, Kim & Prskawetz [36] find a positive effect of shocks on education expenditure. This may be explained by the fact that the death of household members results in allocating the remaining resource in education expenditure for other family members. In addition, some recent studies documented the positive role of social protection program such as health insurance in mitigating shocks including health shock [41]. Indeed, Liu [41] finds that access to health insurance helps households to maintain investment in children’s human capital during negative health shocks, which suggests that one benefit of health insurance could arise from reducing the use of costly smoothing mechanisms. Note in passing that in Chana, Zhang & Gao [76] find that welfare receipt increased health expenditures of older families in rural but have no statistically significant effect in urban China. In addition, it increased expenditures on informal health care. The authors also find that with welfare receipt, less vulnerable households have more self-treatment expenses. It worths mentioning that this article is interested in the short run effect of health shocks.

## A framework for the empirical strategy

The household’s expenditures are reported for the whole household as a single unity of decision. Therefore, we use household level demand function. Household seeks maximize a single utility function. Our framework is based on the conditional demand function formally developed by Pollak [55]. We consider health care as a pre-determined good and use household’s conditional demand for a particular commodity. The households first allocate a certain amount of income on health care as required and then allocate the rest of the income on consumption of others commodities. In presence of pre-determined goods, Pollak [56] shows that the conditional demand function for other goods obtained from the utility maximization of a representative household depend on the prices of these goods ($$p)$$, expenditure on pre-determined good ($${E}_{h})$$, total expenditure on goods other that health care ($${E}_{-h}) and$$ characteristics of the household ($$X)$$. Conditional on the consumption of the health care, the demand function of the other goods can be written as.

1 $${q}_{j}=f\left(p, {E}_{h},{E}_{-h},X\right)$$

where ${q}_{j}$ is the conditional demand function of any given good $j$. With *j* = *1……0.7*

Before moving to the econometrics issues related to the estimation of Eq. (1) we perform a mean comparison test to find out whether OOP health expenditure cause significance difference in the consumption of households with and without OOP medical spending.

Turning to the empirical implementation of the Eq. (1), we first follow some previous studies [51] by using a test developed by Vermeulen [67] to test whether health care users and non- users’ households have different preferences. The data set show a large number of zeros or missing values against the health expenditures. This can be either because health care prices are currently unaffordable to some of the households due to the constraints in their budget or because of abstention. Abstention means that the actual cause of zeros is the heterogeneity in preferences between health spending and non-health households. Vermeulen [67] test consists of augmenting the conditional demand function with a dummy variable $H$ which indicates the status of health care consumption. If this conditioning dummy variable is significant in the demand for the other commodities for all households, we can conclude that health care users and non-users behave differently. In other words, consuming and non-consuming health care households have different preferences and zero OOP health expenditure are not driving from corner solutions. According to Vermeulen [67], the insignificance of this dummy variable is not sufficient to conclude that the zeroes are not caused by abstention in dependent variables. This is also used to test of the weak separability of other commodities from health care [51, 67]. Weak separability means that if a household starts allocating money to health care, this only generates an income effect and no substitution effect on the consumption other goods. The F-statistics for the exclusion of some variables with which the binary indicator H is associated is used for the consumer separability [67].

As cross-sectional data is used, we cannot observe price change. As consequence all households (within geographical area) face same prices. Instead of estimating a demand system as in Eq. (1), we estimate Engel curves for broad commodities to analyze the association between OOP health expenditure and household’s consumption of other goods using the Quadratic Almost Ideal Demand System (QUAIDS) developed by Banks, Blundell, & Lewbel (1996). The QUAIDS model is an example of the empirical demand systems that allows expenditure nonlinearity. Therefore, it allows us to account for any differential effect of OOP health expenditure on household resource allocation for households of a different economic status. The Engel functions is formalized as follows:

2 $${\omega }_{ij}={\beta }_{1i}+{\beta }_{2j}{H}_{i}+{\gamma }_{i}X+({\Psi }_{1j}+{\Psi }_{2j}{H}_{i}){ln{E}_{-H}}_{i}+({\upzeta }_{1j}+{\upzeta }_{2j}{H}_{i}){ln{E}_{-H}}_{i}^{2}+{\mu }_{ij}\sum_{d=1}^{12}{depart}_{i,d}+{\mu }_{ij}$$

where, ${\omega }_{ij}$ is the household *i*’s expenditure share of category *j*. Expenditure shares are calculated after deducting expenditure on health in order to isolate the effect of Out -of pocket health expenditure on budget share for different commodities. ${H}_{i}$ is a participation dummy taking a value of 1 if the household has spent a positive amount on health care, and it allows to account for the discontinuity in the Engel function at zero health expenditure. The interaction terms obtained by combining total non-health expenditure with the incidence of out-of-pocket health expenditure show the marginal effect of OOP health expenditure on budget shares changes with total non-health expenditure.${ln{E}_{-H}}_{i}$ is the natural logarithm of the household consumption expenditure excluding health expenditure. $X is$ is a vector of control variables. ${depart}_{i,d}$ controls for department fixed effects, which takes the value 1 if household i resides in administrative department d, and 0 otherwise. ${\mu }_{ij}$ is the error term. In this study, we consider seven broad categories of consumption items, namely food, education, alcohol, clothing, transportation and communication, housing and other. We estimate conditional Engel curves for six categories, omitting the equation for “other”. The consumer separability is rejected if the parameters associated with the dummy variable $H$ are jointly significant in the Engel curves. Therefore, we can say that expenditure shares allocated towards various commodities are different for households with and without health expenditure. The crowding effect is defined as the marginal effect of OOP health expenditure on expenditure share of a particular consumption category *j.* To account for both the household’s heterogeneity preferences and the level of OOP health expenditure in the estimation of crowding out Eq. 2 can be augmented with the addition of the health expenditure variable, $ln{E}_{Hi}$ as follows:

3 $${\omega }_{ij}=\left({\beta }_{1j}+{\beta }_{2j}{H}_{i}+{\beta }_{3j}{{lnE}_{H}}_{i}+{\gamma }_{i}X\right)+\left({\Psi }_{1j}+{\Psi }_{2j}{H}_{i}+{\Psi }_{3j}{{lnE}_{H}}_{i}\right){ln{E}_{-H}}_{i}+({\upzeta }_{1j}+{\upzeta }_{2j}{H}_{i}+{\zeta }_{3j}{{lnE}_{H}}_{i}){ln{E}_{-H}}_{i}^{2}+\sum_{d=1}^{12}{depart}_{i,d} +{\mu }_{ij}$$

The interaction terms obtained by combining total non-health expenditure with the incidence and level of out-of-pocket health expenditure show the marginal effect of OOP health expenditure on budget shares changes with total non-health expenditure. From Eq. 3, the marginal effect of OOP health expenditure on the budget shares on other categories of good are derived as follows.

4 $$\frac{{\delta \omega }_{ij}}{\delta {lnE}_{{H}_{i}}}={\beta }_{3j}+{\Psi }_{3j}{lnE}_{{-H}_{i}}+{\zeta }_{3j}{ln{E}_{-H}}_{i}^{2}$$

Equation (4) shows that the marginal effect of OOP health expenditure on the consumption of category $j$ depends only on total non-health expenditure. OOP health expenditure crowd out consumption of category $j$ if $\frac{{\delta \omega }_{ij}}{\delta {lnE}_{{H}_{i}}} <0$. Inversely, OOP health expenditure crowd in consumption of category $j$ if $\frac{{\delta \omega }_{ij}}{\delta {lnE}_{{H}_{i}}} >0$. OOP health expenditure has no direct income effect in consumption of category $j$ if $\frac{{\delta \omega }_{ij}}{\delta {lnE}_{{H}_{i}}}=0$.

The challenge in this study is to estimate the crowding out effect of health expenditure on other consumption items. Several techniques have been used in recent studies to investigate the crowding out effect is social sciences. These include the instrumental variables (John, et al., 2011; San and Chaloupka, 2015; Hussain et al., 2018); the Quadratic AIDS model [53] and the Seemingly Unrelated Regression Equation (SURE) technique [52].

Therefore, the system in Eqs. 2 and 3 will be estimated with a Seemingly Unrelated Regression Equation (SURE) technique because all the equations for j categories are simultaneously estimated, as there could be correlation among the error terms of these equations. However, there may appear a potential endogeneity of total non-health expenditure since it may be wrongly measured or jointly driving by the expenditure shares on different categories of goods. For example, the AGVSA data report information on some consumption expenditure with a reference period of 30 days. The small interval of the reference period may explain that Expenditure on certain consumption head may not be positive. Also, zero expenditure does not necessarily mean that households do not consume these goods and services. This infrequency of purchase lead to a measurement error in non-health expenditure. Thus, total expenditure is correlate with the error term. Therefore, the instrumental variable (IV) method is used to cut the correlation between total health expenditure and total non-health expenditure and obtain consistent and unbiased estimators [33]. Studies estimating a conditional demand system use total expenditure as instrument for group expenditure [17, 51]. We use total household budget including out-of-pocket health expenditure as an instrument for total non-health expenditure. Total expenditure is linked to access saving and credit by the households. So, an indication of access to saving and credit could be used as instruments. For a given initial income, a household that has opportunity to save and borrow has a greater capacity to expend their expenditure. In the case where total non-health expenditure is an endogenous variable, 3SLS method is more efficient than SURE to estimate a system of equations. We compared the estimate of 3SLS to those of SURE using Hausman test. In the case where estimates of 3SLS are more efficient than those of SURE, Hausman test will reject the null hypothesis, thus proving the endogeneity of total non-health expenditure.

## Data and descriptive statistics

### Data

Data are drawn from the Global Analysis of Vulnerability and Food Security database (GVFSA/AGVSA). The GVFSA is a survey conducted by the National Institute of Statistics of Benin, with support from the World Food Program (WFP) and other partners. It aims to understand food security in Benin. The survey covered a sample of 14,952 households with 6856 from urban area against 8096 from rural area. It was conducted between over July–August 2017. The WFP (2017) survey is representative at the country level, municipal level and by place of residence. This sample was drawn according to a two-stage sampling design, with a margin of error of 5% (WFP, 2017). At the first level, 750 clusters were drawn from the 920 clusters, then in the second degree, 20 households were drawn, so systematic, in each cluster. The sample was drawn by urban/rural stratum at the level of each commune. A total of 148 strata have been defined in this way. The sample households were distributed in each department proportionally their size in number of households.

The data set includes information at the household level relative to socio-economic characteristics of the household, expenditure on health, education, food, clothing, transport and communication items as well. Since existing literatures listed multiple factors that impacted household consumption patterns [7, 11, 77], the following control variables were used: household demographic characteristics namely, age and the square of age considering the possible non-linear influence, sex, marital status, education years,household characteristics such as family size, children's dependency ratio, and the elderly's dependency ratio. Household resource variables including household assets and income, considering the possible non-linear influence, the assets and income were transformed to logarithms.

### Socio-economic characteristics of households

Table 1 presents socio economic characteristic of households. The derivation of the variables related to health shock is from the following question in the survey. The respondent was asked whether, during the past four weeks, he had been sick, injured, or suffering from a chronic or acute disease; if the response is yes then he is considered as exposed to a health shock. In Benin like in other developing countries, households face a number of shocks including idiosyncratic and covariate shocks. Over the surveyed sample, 44% recorded being exposed to shocks during the four-pass weeks for which 35.88% were idiosyncratic health shocks. In addition, a quasi-totally (94.47%) of those exposed household indicate that shock occurrence reduced their income and consumption pattern. Consequently, only 9.67% of household has been totally recovered from shock. However, 55.64% declared being partially recovery while 33.49% did not. Besides, only 7.38% of households reported having benefited of assistance either from government, NGOs, UN agency, local authority, friends or family member (Table 1). In average, there are about 2 household members that contribute to the household income. The average age of the members of the household is 47 years and the number of spouses for male head of the household is approximately 2.

**Table 1:** Socio economic characteristics

Variable	Obs	Mean	Std.dev	Min	Max
Age	14,952	47.105	14.993	16	100
Contributed to income	14,867	1.587	1.265	0	99
**Categorical Variables**	**Freq**		**Percent (%)**		
Exposure to shocks					
No	8353		56.00		
Yes	6564		44.00		
*Nature of the shock*					
Covariate shock	4209		64.12		
Idiosyncratic health shock	2355		35.88		
The shock Reduced Income					
No	360		5.53		
Yes	6153		94.47		
**Recovered from shock**					
No	2181		33.49		
Partially	3624		55.64		
Entirely	630		9.67		
**Social Assistance**					
No	13,848		92.62		
Yes	1104		7.38		
Poverty status					
Poor	5281		35.32		
Non-poor	9671		64.68		
**Residence**					
Rural	8096		54.15		
Urban	6856		45.85		
**Gender**					
Female	3020		20.20		
Male	11,932		79.80		
**Marital status**					
Single	568		3.80		
Married	12,339		82.52		
Divorced	480		3.21		
Widow	1565		10.47		
**Household composition**					
Presence of child	12,346		82.6		
Presence of older	3611		24.2		
Presence of child and older	2410		16.1		
**Education**					
Uneducated	7659		51.22		
Primary	3880		25.95		
Secondary	2548		17.04		
Superior	885		5.78		

Source: Authors, 2023

Besides, 54.15% of the respondents are in rural area, 79.80% are male and 82.52% are married. Moreover, most of the respondents are uneducated with only 22.82% that have achieved at least a secondary education level.

## Results and discussion

**Fig. 2:**
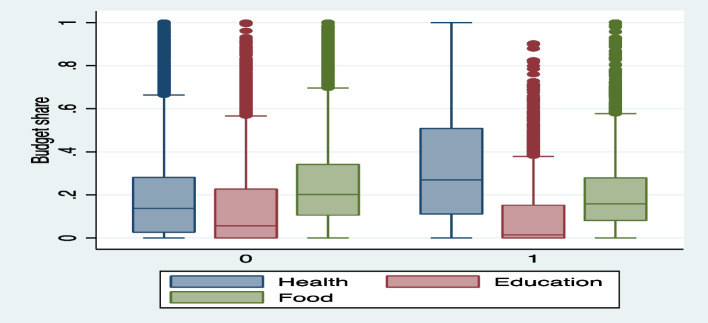
Figure A1: Health shock effect on health, education and food

In the presence of shocks, households tend to use a number of strategies to insure consumption against shocks. These strategies include mainly migration, remittances, increasing labor supply, sale of non-land non-productive assets, increasing borrowing reducing educational and food expenditure among other. We evidenced those strategies by performing first a mean comparison test to compare the average share of consumption items among shocked and non-shocked households in Benin (Table 2, Fig. 2 in appendix). It shows how average consumption type varies between households at risk or not to health shocks. In fact, households exposed to health shock spent significantly more on health care and significantly less on food, education, and clothing as well as transport and communication items.

**Table 2:** Mean differences in expenditure according to shock status

Variables	Full sample		Exposure to health shock				
			No		Yes		t-test (Mean Diff)
	Obs	Mean	Obs	Mean1	Obs	Mean2	
Health expenditure	14,952	80,190	12,021	68,000	2931	130,000	-6.3e + 04***
Education expenditure	14,952	67,796	12,021	71,000	2931	53,000	1.9e + 04***
Food expenditure	14,952	450,995	12,021	460,000	2931	430,000	3.0e + 04***
Cereal	14,952	0.961	12,021	0.959	2931	0.970	-0.011***
Fruit	14,952	0.0804	12,021	0.0880	2931	0.0500	0.038***
Meat	14,952	0.277	12,021	0.294	2931	0.205	0.089***
Fish	14,952	0.713	12,021	0.717	2931	0.699	0.018*
Milk	14,952	0.225	12,021	0.242	2931	0.153	0.089***
Edge	14,952	0.156	12,021	0.169	2931	0.102	0.067***
Alcohol expenditure	14,952	10,091	12,021	10,000	2931	10,000	-103.1
Clothing expenditure	14,952	32,443	12,021	34,000	2931	27,000	7276.682**
Transport & Communication	14,952	148,677	12,021	150,000	2931	130,000	2.4e + 04***
Housing expenditure	14,952	48,559	12,021	54,000	2931	27,000	2.7e + 04***
Other expenditure	14,952	177,087	12,021	180,000	2931	160,000	16,000

Source: Authors, 2023

The result shows a statistically significant difference in the household expenditure between household with health shock and those without health shocks. For example, there are notable differences in those expenditures regarding health status of the household. A part from alcohol and other expenditures, all other expenditures are significant in mean difference.

In average, households spend 80,190 FCFA (146 USD) in health care, 67,796 FCFA (123 USD) in education, 450,995 FCFA (820 USD) in food, 32,443 FCFA (59 USD) in clothing, 10,091 FCFA (18 USD) in alcohol, 148,677 FCFA (270 USD) in transport and communication, 48,559 FCFA (88 USD) in house renting and 78,711 FCFA (143 USD) in other goods. Globally, health and education expenditure following food expenditure count for a big part in the household consumption budget in Benin. Table 3 shows that households that are exposed to an idiosyncratic health shock tend to spend twice more than those that are not. This difference is about 62,000 FCFA (113 USD) and is significant at 1% level. On contrary, the households exposed to health shock spend less in education and food compared with non-exposed household. The resulting differences are 18,000 FCFA (33 USD) and 30,000 FCFA (55 USD) for education and food expenditure respectively. For the latter, it may have implications for the nutrition intake of households. Households exposed to health shock allocate more of their budget to cereals and cereal substitutes. In contrast, cereal, fruit, meat, edge and milk, items mostly consumed by children, are highly compromised among households. Indeed, in the presence of shock, households use several coping strategies including reducing major item spending in the budget especially education and food expenditures. This is more acute in the context of African countries and particularly in Benin where although there is a social protection programs through the ministry of social affair and microfinance to help poor cope shocks, the action is not always prompt to have immediate effect on vulnerable people.

**Table 3:** Crowding out effect of health expenditure

	(1)	(2)	(3)	(4)	(5)	(6)
VARIABLES	Education	Food	Clothing	Alcohol	Trans & com	Housing
OOP health expenditure	-0.094**	-0.029***	-0.003	0.021	-0.128**	-0.065*
	(0.041)	(0.086)	(0.025)	(0.016)	(0.059)	(0.035)
Total expenditure	-0.081	6.764***	-1.963***	-0.230	-1.399	-2.213***
	(0.793)	(1.649)	(0.476)	(0.314)	(1.142)	(0.666)
Total expenditure square	-0.001	-0.248***	0.072***	0.008	0.056	0.084***
	(0.029)	(0.061)	(0.018)	(0.012)	(0.042)	(0.025)
Total expenditure_ OOP health exp	0.015**	-0.046***	0.002	-0.004	0.020**	0.010*
	(0.006)	(0.013)	(0.004)	(0.002)	(0.009)	(0.005)
Total expenditure square _ OOP health exp	-0.001**	0.002***	-0.000	0.000*	-0.001**	-0.000*
	(0.000)	(0.001)	(0.000)	(0.000)	(0.000)	(0.000)
Pro_OOP Expenditure	-0.442	50.151***	-16.489***	-2.633	-8.903	-16.895***
	(6.680)	(13.889)	(4.008)	(2.644)	(9.614)	(5.613)
Pro_OOP health exp_Total _expenditure	-0.133	-7.545***	2.380***	0.369	1.713	2.685***
	(0.991)	(2.060)	(0.594)	(0.392)	(1.426)	(0.833)
Pro_OOP health exp_Total _expenditure square	0.010	0.274***	-0.087***	-0.013	-0.068	-0.102***
	(0.037)	(0.076)	(0.022)	(0.015)	(0.053)	(0.031)
**Control variables**	YES	YES	YES	YES	YES	YES
Constant	1.431	-43.844***	13.605***	1.735	7.295	13.929***
	(5.347)	(11.118)	(3.208)	(2.117)	(7.697)	(4.494)
Observations	11,896	11,896	11,896	11,896	11,896	11,896
R-square	0.104	0.238	0.066	0.063	0.141	0.264

^***^ *p* < 0.01, ** *p* < 0.05, * *p* < 0.1,

Since the mean differences could also be caused by other factors, a multivariate analysis is carried out further to understand the impact of health expenses on consumption items of households. The multivariate analysis methods of SURE and 3SLS are used to estimate Engel curves for each of the seven categories of commodities. We performed Hausman specification test to compare the efficiency of estimates of SURE and 3SLS regressions (Table 4 in appendix). In the case where Hausman test reject the null hypothesis estimates of 3SLS is more efficient, total non-health expenditure is endogenous in nature. Otherwise, estimates of SURE will be more efficient. Results of Hausman test show that SURE could not be rejected as consistent estimator at 1% per cent level of significance. With p-value higher than significant level, Hausman test could not reject the null hypothesis, and thus proves that total non-health expenditure is not endogenous. Therefore, SURE is carried out as the estimation by 3SLS is less efficient than SURE when expenditure is exogenous. We test endogeneity of total non-health expenditure (Table 5 in appendix). Also, the test of consumer separability shows that the share spent on food is higher for poor households who spend on health care (Tables 6 in appendix). This indicates that if households are forced to reduce non-health expenditure to accommodate health expenditure, then they reallocate shares of commodities within non-health expenditure to protect the consumption of necessities.

**Table 4:** Test de Hausman SURE vs 3SLS (Eq. 3)

**Chi2**	698.07
*p*. value	0.0000

**Source**: Authors, 2022

**Table 5:** Endogeneity test

Variables	Durbin (score) chi2(2)	Wu-Hausman F(2,11,904)
Education	13.1386 (0.0014)	6.55999 (0.0014)
Food	35.6586 (0.0000)	17.8378 (0.0000)
Clothing	7.27516 (0.0263)	3.63065 (0.0265)
Alcohol	2.61476 (0.2705)	1.30438 (0.2714)
Transport and communication	62.7152 (0.0000)	31.444 (0.0000)
Housing	52.7152 (0.0000)	24.446 (0.0000)

Sources: Authors, 2022

**Table 6:** Wald test for consumer separability

Variables	Wald chi2 (3)
Education	1.17 (0.7607)
Food	33.35 (0.0000)
Clothing	24.15 (0.0000)
Alcohol	3.09 (0.3786)
Transport and communication	89.08 (0.0000)
Housing	17.03 (0.0007)

Sources: Authors, 2023

Table 3 presents the multivariate analysis of the QUAIDS model using six categories of consumption items. The table displays the crowding out effect of health expenditure on other consumption items including education and food. We reported only the coefficients associated to health expenditure, total expenditure and total expenditure square because our objective is only to analyze the crowding out effect of health expenditure and not the effect of other control variables. The coefficients on education, transport and housing are significantly negative while the others are either positive or insignificant. This finding is in line with the existing literature [47],Dercon et al., 2005) but opposes the work of Panikkassery [52] in some points. In fact, Panikkassery [52] finds for the special case of India that as out of pocket health expenditure increases, household tend to increase their consumption of food and reduce the non-food consumption. Escobal et al. [21] show that shocks negatively affect household education expenditure in Peru. In fact, households compensate the health care expenses by reducing the consumption of other items like, education, food, and housing that count for a big part in the household budget.

The relative share of most of the main expenditure were reduced to accommodate the increase in health expenses. This shows strong coping strategy behavior in terms of consumption modification of households. Food accounts for the largest share of total household expenditure in low-income countries [75]. Lack of access to food may have detrimental effects on health, well-being and labor productivity of households. Since education is also affected in the process of consumption modification, this can result in long-term impacts of the human capital and future living standards of the households. Indeed, the reduction in education expenses as a result of a higher health expenses shows the requirement for more public expenditure for health and education. This because spending on education is perceived as an investment in human capital of households [38] reflecting households' willingness to improve their labor productivity and enhance their well-being in the future. The reduction education expenditure with higher health expenses among households shows the urgency for substantial public expenditure for health and education and the urgency of social protection programs. Consequently, it is important to identify the relevant institutions that enable households to insure against health shocks in developing countries where most of markets are incomplete. As suggested by Vo & Van [68], health insurance can help households reduce the unexpected financial loss from health care and can also reduce losses in human capital from going without medical treatment. The value of health insurance is substantial because it helps household not to reduce educational investment in children in order to smooth consumption against health shocks [15, 41]. In other words, access to health insurance completely mutes the effects of the health shock on the investment in children’s education. Higher health expenditure might change the household consumption pattern and jeopardize the sustainability of some vital goods such as education.

As a robustness check, we estimated the same regressions on the sample of poor and non-poor household. We found that, the crowding out effect may vary among poor and non-poor household (Tables 7 and 8 in appendix). The finding that the budget share of health care increased when an individual in a household is exposed to health shock whilst the other budget shares decrease, is an indication that households, beyond behaving rationally are very vulnerable and need assistance to smooth their consumption. Indeed, the impact of health shocks on consumption and the ability of households (and other risk sharing institutions) to smooth consumption can vary from one item to another. Skoufiasl & Quisumbing (2005) find that adjustments in non-food consumption can act as a mechanism for partially insuring food consumption from the effects of income changes. The results show that irrespective of economic class and public health expenditure, households tend to fund their health expenses by reducing other consumption items mainly food and education items.

**Table 7:** Crowding out effect of health expenditure with control variables

	(1)	(2)	(3)	(4)	(5)	(6)
VARIABLES	Education	Food	Clothing	Alcohol	Trans & com	Housing
OOP health expenditure	-0.094**	-0.029***	-0.003	0.021	-0.128**	-0.065*
	(0.041)	(0.086)	(0.025)	(0.016)	(0.059)	(0.035)
Total expenditure	-0.081	6.764***	-1.963***	-0.230	-1.399	-2.213***
	(0.793)	(1.649)	(0.476)	(0.314)	(1.142)	(0.666)
Total expenditure square	-0.001	-0.248***	0.072***	0.008	0.056	0.084***
	(0.029)	(0.061)	(0.018)	(0.012)	(0.042)	(0.025)
Total expenditure_ OOP health exp	0.015**	-0.046***	0.002	-0.004	0.020**	0.010*
	(0.006)	(0.013)	(0.004)	(0.002)	(0.009)	(0.005)
Total expenditure square _ OOP health exp	-0.001**	0.002***	-0.000	0.000*	-0.001**	-0.000*
	(0.000)	(0.001)	(0.000)	(0.000)	(0.000)	(0.000)
Pro_OOP Expenditure	-0.442	50.151***	-16.489***	-2.633	-8.903	-16.895***
	(6.680)	(13.889)	(4.008)	(2.644)	(9.614)	(5.613)
Pro_OOP health exp_Total _expenditure	-0.133	-7.545***	2.380***	0.369	1.713	2.685***
	(0.991)	(2.060)	(0.594)	(0.392)	(1.426)	(0.833)
Pro_OOP health exp_Total _expenditure square	0.010	0.274***	-0.087***	-0.013	-0.068	-0.102***
	(0.000)	(0.001)	(0.000)	(0.000)	(0.000)	(0.000)
Age	0.001***	0.001***	-0.000***	0.000	-0.001***	0.000
	(0.000)	(0.000)	(0.000)	(0.000)	(0.000)	(0.000)
Gender	-0.012***	-0.030***	-0.004**	0.013***	0.052***	-0.007***
	(0.003)	(0.005)	(0.002)	(0.001)	(0.004)	(0.002)
**Education (No education as reference)**						
1.Primary	0.020***	0.006	0.007**	0.000	-0.039***	-0.009*
	(0.006)	(0.012)	(0.003)	(0.002)	(0.008)	(0.005)
2.Secondary	0.026***	-0.013	0.005*	-0.005**	-0.027***	0.015***
	(0.005)	(0.011)	(0.003)	(0.002)	(0.007)	(0.004)
3.University	0.014***	-0.065***	-0.004*	-0.008***	0.043***	0.054***
	(0.004)	(0.009)	(0.002)	(0.002)	(0.006)	(0.003)
**Household composition**						
Older	-0.034***	0.044***	-0.004	0.001	-0.016**	0.008**
	(0.004)	(0.009)	(0.003)	(0.002)	(0.006)	(0.004)
children	0.019***	0.005	-0.003	-0.003**	-0.010**	-0.001
	(0.003)	(0.007)	(0.002)	(0.001)	(0.005)	(0.003)
child_elder	0.025***	-0.031***	0.005*	-0.001	0.012*	-0.008**
	(0.005)	(0.010)	(0.003)	(0.002)	(0.007)	(0.004)
social_assistance	-0.001	0.017***	-0.004**	-0.001	-0.025***	0.003
	(0.003)	(0.006)	(0.002)	(0.001)	(0.004)	(0.003)
residence	0.006***	-0.033***	0.001	-0.002***	0.013***	0.032***
	(0.002)	(0.004)	(0.001)	(0.001)	(0.003)	(0.001)
1.Migration	0.001	0.009	-0.008***	-0.001	-0.018***	-0.000
	(0.004)	(0.009)	(0.002)	(0.002)	(0.006)	(0.003)
Member_contr_income	0.001	0.004**	-0.000	-0.000	-0.002*	-0.002***
	(0.001)	(0.002)	(0.000)	(0.000)	(0.001)	(0.001)
1.Married	-0.000	-0.017*	-0.008***	0.001	0.012*	-0.009**
	(0.005)	(0.010)	(0.003)	(0.002)	(0.007)	(0.004)
2.Divorced	0.004	-0.012	-0.009**	0.005*	-0.001	-0.013**
	(0.007)	(0.014)	(0.004)	(0.003)	(0.009)	(0.006)
3.Widow	-0.007	-0.005	-0.010***	0.002	0.012	-0.018***
	(0.006)	(0.012)	(0.003)	(0.002)	(0.008)	(0.005)
**Department**						
2. Atacora	0.009**	0.024***	-0.027***	0.015***	0.007	-0.002
	(0.004)	(0.008)	(0.002)	(0.001)	(0.005)	(0.003)
3. Atlantique	0.027***	-0.081***	-0.028***	0.016***	0.022***	0.016***
	(0.004)	(0.008)	(0.002)	(0.002)	(0.006)	(0.003)
4. Borgou	0.007**	-0.009	-0.017***	0.006***	0.022***	0.004
	(0.004)	(0.007)	(0.002)	(0.001)	(0.005)	(0.003)
5. Collines	0.034***	-0.113***	-0.014***	0.007***	0.047***	0.014***
	(0.004)	(0.009)	(0.002)	(0.002)	(0.006)	(0.003)
6. Couffo	0.021***	-0.103***	-0.028***	0.025***	0.050***	-0.004
	(0.004)	(0.008)	(0.002)	(0.002)	(0.006)	(0.003)
7. Donga	0.022***	-0.039***	0.004	0.002	-0.013**	0.011***
	(0.004)	(0.009)	(0.003)	(0.002)	(0.006)	(0.004)
8. Littoral	0.049***	-0.119***	-0.030***	0.005***	0.006	0.088***
	(0.004)	(0.009)	(0.003)	(0.002)	(0.006)	(0.004)
9. Mono	0.027***	-0.049***	-0.029***	0.016***	0.018***	-0.007*
	(0.004)	(0.009)	(0.003)	(0.002)	(0.006)	(0.004)
10. Ouémé	0.019***	-0.090***	-0.030***	0.006***	0.042***	0.029***
	(0.004)	(0.008)	(0.002)	(0.001)	(0.005)	(0.003)
11. Plateau	0.007*	-0.050***	-0.022***	0.004***	0.080***	-0.005
	(0.004)	(0.008)	(0.002)	(0.002)	(0.006)	(0.003)
12. Zou	0.002	-0.127***	-0.037***	0.015***	0.086***	0.008**
	(0.004)	(0.008)	(0.002)	(0.001)	(0.005)	(0.003)
Constant	1.431	-43.844***	13.605***	1.735	7.295	13.929***
	(5.347)	(11.118)	(3.208)	(2.117)	(7.697)	(4.494)
Observations	11,896	11,896	11,896	11,896	11,896	11,896
R-Square	0.104	0.238	0.066	0.063	0.141	0.264

**Source**: Authors, 2022

**Table 8:** Crowding out effect of health expenditure with control variables (Non Poor households)

	(1)	(2)	(3)	(4)	(5)	(6)
VARIABLES	Education	Food	Clothing	Alcohol	Trans & com	Housing
OOP health expenditure	-0.295***	-0.209	-0.120**	-0.083*	0.632***	0.173*
	(0.113)	(0.218)	(0.059)	(0.042)	(0.154)	(0.096)
Total expenditure	-1.839	-1.627	-1.099	-0.335	3.419*	-1.866
	(1.422)	(2.755)	(0.749)	(0.535)	(1.939)	(1.216)
Total expenditure square	0.062	0.053	0.040	0.011	-0.118*	0.073*
	(0.051)	(0.099)	(0.027)	(0.019)	(0.070)	(0.044)
Total expenditure_ OOP health exp	0.044***	0.030	0.017**	0.011*	-0.091***	-0.024*
	(0.016)	(0.032)	(0.009)	(0.006)	(0.022)	(0.014)
Total expenditure square _ OOP health exp	-0.002***	-0.001	-0.001*	-0.000	0.003***	0.001
	(0.001)	(0.001)	(0.000)	(0.000)	(0.001)	(0.001)
Pro_OOP Expenditure	-13.728	-11.681	-7.525	-1.944	20.908	-17.231*
	(11.964)	(23.175)	(6.298)	(4.497)	(16.314)	(10.228)
Pro_OOP health exp_Total _expenditure	1.690	1.391	1.098	0.271	-2.627	2.814*
	(1.727)	(3.345)	(0.909)	(0.649)	(2.355)	(1.476)
Pro_OOP health exp_Total _expenditure square	-0.055	-0.045	-0.041	-0.010	0.088	-0.108**
	(0.062)	(0.121)	(0.033)	(0.023)	(0.085)	(0.053)
Age	0.001***	0.001***	-0.000***	0.000	-0.001***	0.000
	(0.000)	(0.000)	(0.000)	(0.000)	(0.000)	(0.000)
Gender	-0.017***	-0.017***	-0.005***	0.012***	0.049***	-0.011***
	(0.003)	(0.006)	(0.002)	(0.001)	(0.004)	(0.003)
**Education (No education as reference)**						
1.Primary	0.036***	-0.012	0.004	0.000	-0.032***	-0.013**
	(0.007)	(0.013)	(0.004)	(0.003)	(0.009)	(0.006)
2.Secondary	0.043***	-0.028**	0.003	-0.005**	-0.023***	0.011**
	(0.006)	(0.012)	(0.003)	(0.002)	(0.008)	(0.005)
3.University	0.014***	-0.065***	-0.000	-0.007***	0.036***	0.064***
	(0.005)	(0.009)	(0.003)	(0.002)	(0.007)	(0.004)
**Household composition**						
older	-0.040***	0.029***	-0.004	0.000	-0.004	0.014***
	(0.005)	(0.010)	(0.003)	(0.002)	(0.007)	(0.004)
children	0.014***	0.013*	-0.001	-0.002	-0.010*	0.002
	(0.004)	(0.007)	(0.002)	(0.001)	(0.005)	(0.003)
child_elder	0.027***	-0.019*	0.005*	0.000	0.003	-0.013***
	(0.005)	(0.011)	(0.003)	(0.002)	(0.007)	(0.005)
social_assistance	-0.001	0.006	-0.001	-0.001	-0.025***	0.004
	(0.004)	(0.007)	(0.002)	(0.001)	(0.005)	(0.003)
residence	0.005**	-0.030***	-0.001	-0.002**	0.013***	0.035***
	(0.002)	(0.004)	(0.001)	(0.001)	(0.003)	(0.002)
1.Migration	0.004	-0.001	-0.003	-0.002	-0.018**	0.004
	(0.005)	(0.010)	(0.003)	(0.002)	(0.007)	(0.005)
Member_contr_income	0.000	0.004*	-0.002***	-0.001	-0.001	-0.002**
	(0.001)	(0.002)	(0.001)	(0.000)	(0.002)	(0.001)
**Marital status (Single as reference)**						
1.Married	-0.002	-0.019*	-0.002	0.000	0.011	-0.012**
	(0.006)	(0.011)	(0.003)	(0.002)	(0.008)	(0.005)
2.Divorced	0.005	-0.016	-0.007*	0.006**	-0.001	-0.017***
	(0.008)	(0.015)	(0.004)	(0.003)	(0.010)	(0.006)
3.Widow	-0.009	-0.023*	-0.004	0.002	0.015*	-0.020***
	(0.007)	(0.013)	(0.004)	(0.003)	(0.009)	(0.006)
**Departments**						
2. Atacora	0.004	0.029***	-0.035***	0.012***	0.041***	-0.009*
	(0.005)	(0.011)	(0.003)	(0.002)	(0.007)	(0.005)
3. Atlantique	0.029***	-0.084***	-0.035***	0.010***	0.043***	0.016***
	(0.005)	(0.010)	(0.003)	(0.002)	(0.007)	(0.004)
4. Borgou	0.011**	0.003	-0.027***	0.004**	0.029***	-0.002
	(0.005)	(0.010)	(0.003)	(0.002)	(0.007)	(0.004)
5. Collines	0.046***	-0.149***	-0.016***	0.005**	0.079***	0.005
	(0.006)	(0.011)	(0.003)	(0.002)	(0.008)	(0.005)
6. Couffo	0.017***	-0.096***	-0.034***	0.024***	0.061***	-0.008*
	(0.005)	(0.010)	(0.003)	(0.002)	(0.007)	(0.005)
7. Donga	0.019***	-0.057***	-0.012***	0.001	0.012	0.007
	(0.007)	(0.013)	(0.003)	(0.002)	(0.009)	(0.006)
8. Littoral	0.054***	-0.123***	-0.035***	0.003*	0.023***	0.083***
	(0.005)	(0.010)	(0.003)	(0.002)	(0.007)	(0.005)
9. Mono	0.030***	-0.063***	-0.032***	0.013***	0.036***	-0.011**
	(0.005)	(0.011)	(0.003)	(0.002)	(0.007)	(0.005)
10. Ouémé	0.025***	-0.089***	-0.035***	0.005***	0.056***	0.021***
	(0.005)	(0.009)	(0.003)	(0.002)	(0.007)	(0.004)
11. Plateau	0.009*	-0.072***	-0.025***	0.004*	0.108***	-0.009*
	(0.005)	(0.010)	(0.003)	(0.002)	(0.007)	(0.005)
12. Zou	0.001	-0.120***	-0.044***	0.014***	0.091***	0.007
	(0.005)	(0.010)	(0.003)	(0.002)	(0.007)	(0.004)
Constant	14.118	13.975	7.644	2.470	-25.766*	11.093
	(9.846)	(19.072)	(5.183)	(3.701)	(13.426)	(8.418)
Observations	8,082	8,082	8,082	8,082	8,082	8,082
R-squared	0.133	0.255	0.069	0.064	0.140	0.292

## Concluding remarks

In this study we investigated the household response to health shocks using a recent national households survey 14,952 households in Benin. to evidence the crowding out effect of health expenditure on other household’s consumption items. Our findings show that health shocks lead households to behave rationally by reducing their budget share devoting to food and non-food good in order to finance their health care. However, higher health expenditure is detrimental to human capital because of the lack of social protection program that should have the mandate to ensure health care and prevent household from catastrophic health expenditures. Coping strategy by altering consumption also shows the vulnerability of households to the financial shock caused by illness. The lack of proper safety net mechanisms and poor functioning of public health care facilities aggregate their vulnerability.

These findings lead to several policy implications. First, we believe that a better understanding by the public of the importance of human capital including health and education might influence some individuals to never forgone expenditure on education and foods although they have to increase their expenditure on health due to chocs. Therefore, households should be supported in coping with health shocks in order to avoid welfare losses. Therefore, establishment and development of health care systems, including health insurance should be seen as of utmost importance. For instance, a proper outreach of health insurance programs and a well-functioning of public health care facilities can help poor in maintaining the same consumption bundle to a greater extent. However, it may be interesting to investigate in the future researches how to structure the healthcare that can include some of the catastrophic expenditure so that to efficiently assist poor vulnerable people exposed to shocks.

## Data Availability

Available upon request.

## Appendix

Figure 2

Table 4

Table 5

Table 6

Table 7

Table 8

Table

**Table 9:** Crowding out effect of health expenditure with control variables (Poor household)

	(1)	(2)	(3)	(4)	(5)	(6)
VARIABLES	Education	Food	Clothing	Alcohol	Trans & com	Housing
OOP Health expenditure	-0.143**	-0.236	0.048	-0.035	-0.096	0.092
	(0.072)	(0.174)	(0.055)	(0.033)	(0.118)	(0.057)
Total expenditure	-0.633	10.466***	-3.585***	-0.435	-1.558	0.255
	(1.613)	(3.878)	(1.238)	(0.728)	(2.628)	(1.283)
Total expenditure square	0.018	-0.392**	0.138***	0.013	0.064	-0.007
	(0.064)	(0.154)	(0.049)	(0.029)	(0.104)	(0.051)
Total expenditure_ OOP health exp	0.022*	0.041	-0.006	0.005	0.013	-0.015
	(0.012)	(0.029)	(0.009)	(0.005)	(0.020)	(0.010)
Pro_OOP Expenditure	-4.123	84.307***	-29.551***	-4.194	-8.800	1.615
	(12.779)	(30.728)	(9.812)	(5.772)	(20.822)	(10.169)
Pro_OOP health exp_Total _expenditure	0.552	-13.129***	4.477***	0.545	1.913	-0.151
	(2.033)	(4.888)	(1.561)	(0.918)	(3.312)	(1.618)
Pro_OOP health exp_Total _expenditure square	-0.014	0.494**	-0.172***	-0.017	-0.079	0.002
	(0.081)	(0.194)	(0.062)	(0.037)	(0.132)	(0.064)
Total expenditure square _ OOP health exp	-0.001	-0.002	0.000	-0.000	-0.000	0.001
	(0.001)	(0.001)	(0.000)	(0.000)	(0.001)	(0.000)
age	0.000***	0.000	-0.000***	0.000	-0.001***	0.000
	(0.000)	(0.000)	(0.000)	(0.000)	(0.000)	(0.000)
gender	-0.005	-0.056***	-0.001	0.013***	0.061***	-0.000
	(0.004)	(0.010)	(0.003)	(0.002)	(0.007)	(0.003)
**Education (No education as reference)**						
1.Primary	-0.015	0.044*	0.014*	-0.002	-0.066***	0.009
	(0.009)	(0.023)	(0.007)	(0.004)	(0.015)	(0.008)
2.Secondary	-0.012	0.009	0.010	-0.002	-0.041***	0.027***
	(0.009)	(0.022)	(0.007)	(0.004)	(0.015)	(0.007)
3.University	0.006	-0.036*	-0.019***	-0.006	0.064***	0.004
	(0.009)	(0.021)	(0.007)	(0.004)	(0.014)	(0.007)
**Household composition**						
older	-0.011	0.045*	-0.006	0.004	-0.041***	-0.012
	(0.010)	(0.023)	(0.007)	(0.004)	(0.016)	(0.008)
children	0.027***	-0.017	-0.009	-0.004	-0.012	-0.010
	(0.008)	(0.019)	(0.006)	(0.004)	(0.013)	(0.006)
child_elder	0.008	-0.027	0.007	-0.002	0.028*	0.010
	(0.010)	(0.024)	(0.008)	(0.004)	(0.016)	(0.008)
social_assistance	0.006	0.033***	-0.010**	-0.001	-0.021**	-0.002
	(0.005)	(0.012)	(0.004)	(0.002)	(0.008)	(0.004)
residence	0.009***	-0.034***	0.005**	-0.002	0.011**	0.023***
	(0.003)	(0.007)	(0.002)	(0.001)	(0.005)	(0.002)
1.Migration	-0.009	0.035**	-0.017***	0.001	-0.026**	-0.007
	(0.006)	(0.015)	(0.005)	(0.003)	(0.010)	(0.005)
Member_contr_income	-0.000	0.006**	0.000	0.000	-0.003*	-0.002**
	(0.001)	(0.003)	(0.001)	(0.001)	(0.002)	(0.001)
**Marital status**						
1.Married	-0.007	0.013	-0.032***	0.006	0.015	0.004
	(0.011)	(0.028)	(0.009)	(0.005)	(0.019)	(0.009)
2.Divorced	-0.009	0.015	-0.026**	0.006	-0.001	0.006
	(0.014)	(0.034)	(0.011)	(0.006)	(0.023)	(0.011)
3.Widow	-0.011	0.044	-0.034***	0.005	0.010	-0.003
	(0.012)	(0.029)	(0.009)	(0.006)	(0.020)	(0.010)
**Department**						
2. Atacora	0.011**	0.030**	-0.021***	0.018***	-0.027***	0.003
	(0.005)	(0.012)	(0.004)	(0.002)	(0.008)	(0.004)
3. Atlantique	0.029***	-0.077***	-0.021***	0.029***	-0.002	0.000
	(0.006)	(0.015)	(0.005)	(0.003)	(0.010)	(0.005)
4. Borgou	0.005	-0.030**	-0.005	0.007***	0.020**	0.010***
	(0.005)	(0.012)	(0.004)	(0.002)	(0.008)	(0.004)
5. Collines	0.018***	-0.053***	-0.014***	0.009***	0.004	0.022***
	(0.006)	(0.014)	(0.005)	(0.003)	(0.010)	(0.005)
6. Couffo	0.038***	-0.123***	-0.020***	0.024***	0.048***	-0.000
	(0.006)	(0.014)	(0.005)	(0.003)	(0.010)	(0.005)
7. Donga	0.025***	-0.028**	0.015***	0.004*	-0.034***	0.015***
	(0.005)	(0.013)	(0.004)	(0.002)	(0.009)	(0.004)
8. Littoral	0.007	-0.059**	-0.033***	0.006	-0.021	0.035***
	(0.012)	(0.028)	(0.009)	(0.005)	(0.019)	(0.009)
9. Mono	0.034***	-0.021	-0.031***	0.024***	-0.002	-0.010*
	(0.007)	(0.018)	(0.006)	(0.003)	(0.012)	(0.006)
10. Ouémé	0.012*	-0.105***	-0.032***	0.006*	0.033***	0.045***
	(0.007)	(0.017)	(0.005)	(0.003)	(0.011)	(0.005)
11. Plateau	0.008	0.000	-0.024***	0.003	0.037***	-0.005
	(0.006)	(0.015)	(0.005)	(0.003)	(0.010)	(0.005)
12. Zou	0.011**	-0.143***	-0.032***	0.016***	0.095***	0.001
	(0.005)	(0.013)	(0.004)	(0.002)	(0.009)	(0.004)
Constant	4.477	-66.705***	23.719***	3.363	7.362	-2.093
	(10.149)	(24.404)	(7.793)	(4.584)	(16.537)	(8.076)
Observations	3,814	3,814	3,814	3,814	3,814	3,814
R-squared	0.074	0.140	0.085	0.082	0.139	0.103

^9^

## Footnotes

1 RAMU: Régime d’Assurance Maladie Universelle (Universal Health Insurance Regime).

2 CNSS: Caisse Nationale de Sécurité Sociale (National Fund for Social Security); FNRB: Fonds National de Retraite du Bénin (National Retire Fund of Benin).
